# Flood susceptibility zoning in Botswana’s Notwane Urban Catchment: A triangulated and blended analytical hierarchy process-geographical information systems-based multi-criteria analysis

**DOI:** 10.4102/jamba.v18i1.2013

**Published:** 2026-05-15

**Authors:** Gorata Samuel, Reuben J. Sebego, Ditiro B. Moalafhi, Gagoitseope Mmopelwa

**Affiliations:** 1 Department of Environmental Science, Faculty of Science, University of Botswana, Gaborone, Botswana; 2 Department of Wildlife and Aquatic Resources, Faculty of Natural Resources, Botswana University of Agriculture and Natural Resources, Gaborone, Botswana

**Keywords:** analytical hierarchy process, flood conditioning factors, flood susceptibility mapping, GIS, Notwane catchment

## Abstract

Botswana’s economy is highly exposed to floods, estimated to cost an average of $90 million, about 0.56% of the total annual country gross domestic product. Flooding is common in urban catchments, including Botswana’s Notwane Catchment. The catchment is characterised by increased population anchored in the capital Gaborone, infrastructure expansion and altered natural drainage. The effects of floods necessitate proactive interventions that consider blended approaches for selecting, assessing and mapping flood conditioning factors (FCFs). This study aimed to demonstrate a triangulated, multicriteria approach for flood susceptibility mapping in the Notwane Catchment. The FCFs were identified through questionnaire surveys, key informant interviews and literature, and these included land-use, rainfall, elevation, flow accumulation, slope and soil type, among others. The analytical hierarchy process (AHP), supported by principal component analysis (PCA), was employed to derive the weights of factors using the Business Performance Management Singapore AHP tool in Microsoft Excel. Weighted factors were integrated into ESRI’s ArcMap 10.8 for spatial analysis and mapping. Cumulative rainfall (38.79%) emerged as the most critical factor, while altered land-use (21.35%), with statistically significant clustering of risk around built-up areas and water bodies (GiP-value = 0.062), was the second-largest contributor. Approximately 57.6% (5201.2 km^2^) of the catchment area is at moderate risk of flooding, covering Greater Gaborone, Mochudi, Ramotswa, and Kanye.

**Contribution:**

Research and promotion of triangulated and blended approaches anchored on geospatial technologies to investigate flood susceptibility will influence targeted and site-specific risk-informed development in urban catchments while addressing Goal 11 of the 2030 Agenda for Sustainable Development.

## Introduction

Botswana has not been spared because flooding devastates most parts of the southern African region (UNICEF Botswana 2025). Botswana has experienced noticeable floods in the past few decades, including 1972, 1988, 1995, 2000, 2013, and 2017, as indicated in the Emergency Events Database (EM-DAT) (CRED 2021). Rainfall accumulations increased to more than 200 millimetres in one storm event, and about $285 000 000.00 damage was reported throughout the country during the February 2000 floods (USGS 2002). The Ex-Dineo cyclone floods hit Botswana in a short time between 18 and 23 February 2017, where significant flooding caused damage to human properties and infrastructure (Statistics Botswana 2018). A total of 650 homes were affected, with over 500 houses damaged, whereas infrastructure, telecommunication lines and livelihoods were also affected (International Federation of Red Cross [IFRC] 2017). While flooding in Botswana is not necessarily concentrated only in urban centres, its impacts in urban centres are more severe, considering a lot of infrastructure investment. Population densities are also high in urban centres; thus, damage to property and threats to life are more common. This includes the Gaborone-anchored cluster, which lies in the Notwane Catchment and is the country’s central urban cluster. Pourazar (2017) likewise identified Gaborone as one of the most densely populated towns with increased imperviousness and increased population over the catchment, which were matched by the expansion of infrastructure and altered natural drainage. Du et al. (2015) envision that high-intensity rainfall (resulting from climate change and variability) and rapid land-use change (that is responsible for increased imperviousness) may be the leading causes of flooding in urban catchments. As aforementioned, changing rainfall dynamics and urban growth’s massive land-use changes intensify extreme natural events like floods (Parida, Moalafhi & Kenabatho 2006). Change in rainfall characteristics (amount, intensity, duration, frequency, seasonal distribution) is one of the obvious indicators of climate variability and change. Climate change poses a significant threat to Botswana in that it causes large environmental shocks and stresses, such as flash floods, and these events are likely to exacerbate the frequency, intensity and volatility with persistent warming trends (United Nations Development Programme [UNDP] 2018). The inhabitants of the Notwane Catchment have been frequently affected by floods. A notable example of such floods with devastating effects is the Taung village (about 20 km south of Gaborone City) during the 2017 floods, which left the village stranded on either side of the Taung Bridge (Altchenko et al. 2016). In most occurrences, these floods are because of excess rainfall, where excess water flow is usually unexpected and unplanned for, thus causing damage to property and human life in settlements (Toteng 2018). Previous studies point out that an increase in the imperviousness of surfaces as a result of rapid land-use changes over the catchment area results in shortening of flood concentration time, increased flood peak, increased run-off coefficient during heavy falls and reduced storage capacity of river networks (Vojtek & Vojteková 2016) and reservoirs. This is because of disturbed natural run-off and reduced infiltration, consequently increasing vulnerability of the inhabitants and exposing people and properties to the risk of flooding (Du et al. 2019). Despite the increasing frequency and intensity of flooding in Botswana, flood risk reduction projects are not normally included in development plans and budgets; consequently, flood disaster risk reduction techniques and early warning strategies are underdeveloped (NDMO 2012). This explains the increasing frequency and intensity of flood disasters throughout Botswana, and the resultant impacts since 2000 (Toteng 2018), even though these floods date back to the 1970s and possibly beyond. One of the priorities of the world’s current leading disaster management frameworks, the Sendai Framework, is to encourage the use of technology in disaster management to enhance preparedness, response and resilience through data-driven approaches such as remote sensing, machine learning and Geographical Information Systems (GIS) (United Nations 2015). Flood modelling as a data-driven approach is progressively more relevant given the magnitude of potential loss and disruption associated with riverine flooding (Baky, Islam & Paul 2020; Nkwunonwo, Whitworth & Baily 2020), like in the Notwane Catchment. There is, therefore, a need to invest in more comprehensive vulnerability assessments (Botezan, Radovici & Ajtai 2022) that consider blended and complementary assessment tools leveraging advancements in research innovation and technology within frameworks that address the physical dimension of flooding in urban set-ups characterised by high population density, rapidly changing drainage systems and high infrastructural investments (Chang et al. 2021). According to Ouma and Tateishi (2014), geospatial technologies are the most widely used technology for flood analyses, especially in developed countries, as they produce a visualisation of flooding and create potential for further analysis and estimation of probable damage because of floods. Despite the humongous potential of geospatial technologies in flood management, literature has shown some deficiency in utilising these technologies for flood disasters or disasters in general. Specifically, in Botswana, while most African countries have moved towards managing disasters through advanced technologies, the country has not fully leveraged these technologies at the governmental and scientific levels for future decision-making. Literature sources identify various types of flood risk models developed in a GIS environment, including the Analytical Hierarchy Process (AHP), which originated from Saaty (1977) and is designed to solve multi-criteria decision problems through pairwise comparisons. This method is effective for formulating the representation of a complex problem, measuring priorities and choosing alternatives while assessing consistencies (Ogato et al. 2020; Saaty 1977; Seejata et al. 2018). Ouma and Tateishi (2014) integrated the AHP into a GIS environment to create an urban flood risk index for the Eldoret Municipality in Kenya. This integration serves as a powerful tool for evaluating flooding, enabling consistent and efficient use of spatial data (Lin et al. 2019; Mujib et al. 2021; Ouma & Tateishi 2014). Several other models have been developed based on the AHP, including the analytical network process (ANP), the fuzzy AHP (FAHP) and artificial neural networks (ANN), among others. However, AHP, as a multi-criteria model, can carry some bias and subjectivity, highlighting the necessity for validation. Sensitivity analysis is thus commonly employed in most multi-criteria decisions to validate the models and assess the impact of varying judgements on the stability of the outcomes. By modifying the weights of each key conditioning parameter, the extent of sensitivity in changing scenarios is evaluated (Badea & Bacoţiu 2002). Despite global advocacy from frameworks and advancements in flood risk modelling through the integration of GIS and multi-criteria decision-making tools like AHP, such approaches remain underutilised in Botswana. This study underscores the importance of incorporating local knowledge to enhance model accuracy through community participation, as locals are the first victims of floods. To date, no studies have been undertaken in the Notwane Catchment. Additionally, Botswana has few documented applications of AHP-based GIS modelling for flood susceptibility analysis, including flood maps, creating a critical gap in spatial risk assessment in the country. This study addresses this gap by producing the first flood susceptibility map for the Notwane urban catchment using an innovative AHP-GIS approach, which offers a replicable and evidence-based structure for future flood susceptibility mapping in semi-arid regions with limited data. Therefore, this study aims to develop a spatially explicit flood susceptibility map that demonstrates a triangulated and blended innovative multi-criteria approach over Botswana’s most urbanised catchment for flood susceptibility and risk analysis using the Business Performance Management Singapore Version 2022.07.08 (BPMSG) AHP tool.

## Research methods and design

### Study area

**FIGURE 1:**
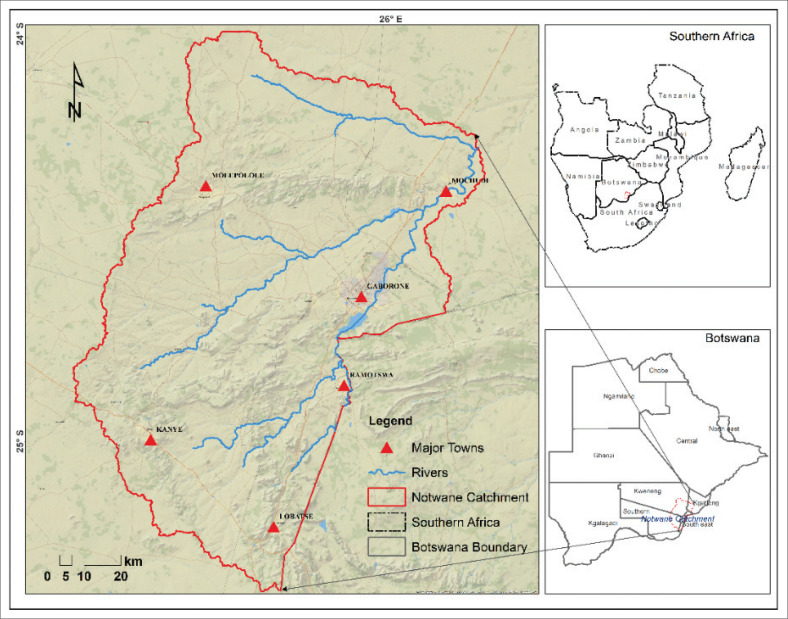
Map of the study area (Notwane Catchment, Botswana).

The Notwane Catchment is located upstream of the Gaborone dam within the Southeast, Southern and Kweneng districts as shown in Figure 1 and within proximity to Gaborone, the capital of Botswana at longitude 25.5°E to 26.0°E and latitude 24.5°S to 25.5°S. The catchment is drained mainly by the ephemeral Notwane River, where it bears its name (Kenabatho, Parida & Moalafhi 2017) and covers about 9260 km^2^ and hosts the country’s largest population cluster (Tafila et al. 2022). The Notwane Catchment is reflective of the Botswana (southeast) climate, which is generally semi-arid with hot summers and dry, cold winters. Long-term average rainfall between January and March (1981–2010) ranges from 80 mm to 340 mm for the whole country (Department of Meteorological Services 2024). The Notwane Catchment lies in the clayey (31% of clay loam Leptosols and 22% of sandy clay loam Acrisols) and sandy river (sandy loam) soils (Franchi et al. 2020). The catchment has witnessed changes in land-use because of growth in settlements and mixed small-scale farming practices (Matlhodi et al. 2019), with a dominance of shrubland and grassland.

### Data collection methods

With limited hydrological and historical flood data availability in the Notwane Catchment, community participation was expedited through structured surveys (key informant interviews and questionnaires) to identify and rate relevant flood conditioning factors (FCFs) for flood susceptibility assessment. A closed-ended questionnaire and interview guide were designed based on Saaty’s scale. Likert scale questions were based on literature identified FCFs and analysed using the BPMSG Version 2022.07.08 AHP software hosted by Microsoft Excel (Goepel 2018) to derive weights and run a principal component analysis (PCA) of the selected FCFs. These factors had unequal degrees of influence, and therefore, the level of impact of each factor on the production of valid susceptibility maps was estimated. The questionnaire was administered to selected participants in the five (5) major settlements and towns within the catchment: Kanye, Lobatse, Greater Gaborone, Ramotswa, Mochudi and Molepolole. Regarding inclusion, participants comprised males and females aged 20–70 years old. Participants who have lived for more than 10 years were included as a result of their understanding of the land-use dynamics in their area. Stratified random sampling using a fishnet and multi-value extraction spatial analyst tools in Arc Map 10.5 were employed to select sample points, determined by the sample size generated through the Yamane (1967) formula (Equation 1) for each major settlement in the catchment area at a 90% confidence level, resulting in a total of 309 respondents. Key informants were selected through an exponential discriminative chain-referral sampling technique based on their expertise and experience to enhance the efficiency of stakeholder identification (Buba et al. 2021). The key players (National Disaster Management Office, Department of Water and Sanitation and Department of Meteorological Services) provided referrals based on the following inclusion and exclusion criteria: (1) Preference was given to officers stationed at each one of the participating sites, and (2) Experts in disaster management and/or those who have experienced historical floods (Dikgosi, village development committees and GIS experts). This approach provided a reliable alternative to historical flood records, which are not adequately documented, ensuring an indigenous and context-specific factor rating for each study site within the catchment for the AHP-GIS model. The AHP was chosen for its ability to evaluate the relative importance of FCFs, and its pairwise comparison capacity made it relevant for flood susceptibility research in data-limited regions, enabling consistent weighting of variables (Arora et al. 2024; Tabasi, Fereshtehpour & Roghani 2025). The decision to use the AHP method was based on its ability to address the problem of inner dependencies between elements in a network (Badea & Bacoţiu 2002). Identified factors based on literature, the questionnaire survey and key informant interviews included land-use and land cover, elevation, slope, soil type, rainfall, flow accumulation, stream networks, and distance from the river, as shown in Table 1, along with their respective metadata for flood mapping.


$$n=N/1+Ne^{2}$$ [Eqn 1]

where *n* = Sample size; *N* = household population; *e* = Margin of error

**TABLE 1:** Summary of datasets used in the study (year, resolution, sources, preprocessing and justification).

Type of data	Year	Spatial resolution	Source	Preprocessing	Justification
Shuttle Radar Topography Mission (SRTM) – Digital Elevation Model (DEM)	2014	1 arc sec (30 m)	https://earthexplorer.usgs.gov/	Hydrologic corrections – filling of sinks, clipping to catchment boundary, derived variables (slope, flow accumulation, stream network and distance to the river) using ArcGIS Pro	Topographic data were essential as it controls surface runoff and accumulation through the identified variables
Soil	1985	1 km	https://www.fao.org/soils-portal/data-hub/soil-maps-and-databases/harmonized-world-soil-database-v20/en/	Downloaded vector layer of soils, clipped to catchment boundary, converted to raster, resampled to 30 m	Soils determine infiltration and runoff
Climate Hazards Group InfraRed Precipitation with Station data (CHIRPS) – rainfall	1981–2020	0.05° (~5.5 km)	https://data.chc.ucsb.edu/products/CHIRPS-2.0/	Clipped to the catchment boundary and resampled to 30 m	Rainfall variability affects flood dynamics
Land cover	2021	10 m	https://worldcover2021.esa.int/	Downloaded, clipped to the catchment boundary and resampled to 30 m	Land use and land cover change affect imperviousness and runoff

The identified factors were rated using Saaty’s scale (1–9) for pairwise comparison, capturing subjective preferences and priorities in a quantifiable manner (rating) from the participating population. The results of the questionnaire and interview surveys were then entered into the BPMSG AHP software to derive weights and consistency ratio (CR) for validation, given that the region has limited historical flood data, and it is heavily reliant on local expertise (Laka et al. 2023; Sofi et al. 2024). A spatial geodatabase was created to incorporate the weighted factors into ESRI’s ArcGIS Pro, where all thematic layers of the conditioning factors were generated to show their level of influence in flood generation. ESRI’s ArcGIS Pro and ArcMap 10.8 were selected mainly for their powerful spatial analysis tools for integrating AHP-derived weights while enhancing the accuracy and usability of flood susceptibility mapping. Each factor was developed into susceptibility maps using reclassification and the Global Getis-Ord Gi spatial statistical tool, which classified them into five levels: very high, high, moderate, low and very low. The very high category represents the most critical susceptibility level, while the very low category indicates the least. The Global Getis-Ord Gi used *z*-scores and *p*-values to define the spatial clustering of FCFs and their spatial significance. Finally, the susceptibility maps were produced using weighted overlay analysis in ArcMap 10.8, and the validation of weights was conducted through sensitivity analysis. Sensitivity analysis utilised weights obtained from the AHP to test the sensitivity of changing criterion weights of each FCF using the ± 20% on baseline weights, a range commonly used to test Multi-Criteria Decision Analysis (MCDA)-based flood models’ sensitivity and robustness (e.g. Kazakis, Kougias & Patsialis 2015). Each factor’s weight was modified through 20% incremental and 20% decremental variations at 5% intervals, all equalling 1. Sensitivity scenarios were then produced to recalculate the sensitivity of the model, in which each weight was quantified by calculating the sensitivity index (SI) using the formula (Equation 2):

SI=Scenario value/Baseline value [Eqn 2]

where SI = 1 no change/variation; SI > 1 decreased variation; SI > 1 increased variation

The blended AHP-GIS approach used in this study is subject to uncertainties and/or limitations because of its heavy reliance on subjective experts and local respondents’ judgement for factor identification and rating, which may introduce biases in results. Limited historical flood data and higher spatial resolution data of the identified FCFs further affect the precision of the analysis. For instance, vector and raster data may have been subjected to accuracy and potential interpolation oversights during preprocessing.

### Ethical considerations

Ethical clearance to conduct this study was obtained from the University of Botswana (Ref. No. UBR/RES/IBR/SOC/172).

## Results

### Selection of key flood conditioning parameters

**FIGURE 2:**
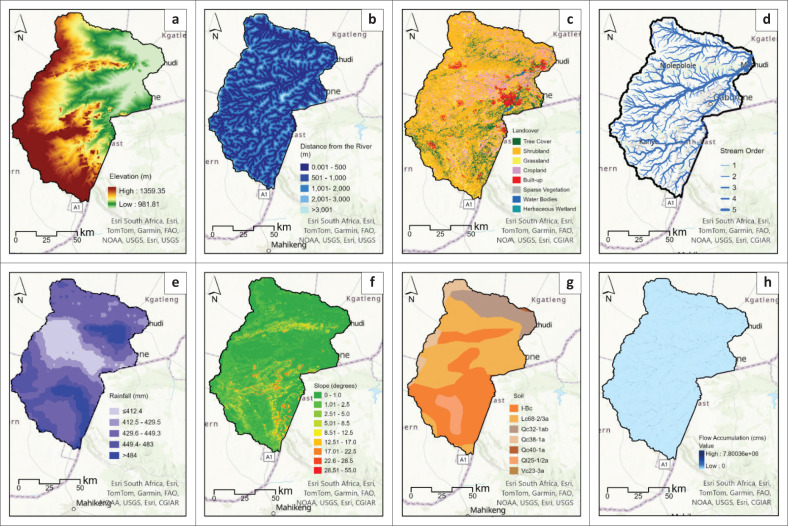
Selected flood conditioning factors (FCFs): (a) elevation, (b) distance from the river, (c) landcover, (d) stream networks, (e) rainfall, (f) slope, (g) soil and (h) flow accumulation.

Figure 2 shows selected FCFs derived from a questionnaire survey, literature and key informant interviews. Elevation has been identified as a contributing factor to flood dynamics, among others. A digital elevation model (DEM) was used to derive various hydrological factors, including flow accumulation, stream order and distance to the river, as well as slope. The DEM indicates that the highest elevation is (1359.35 masl) primarily in the southern and southwestern parts, while the lowest (981.81 masl) is mostly in the northeastern part, as shown in Figure 2a. Distance from the river shown in Figure 2b illustrates areas closer to the river, identified as flood plains (0 m – 1000 m), which are usually susceptible to riverbank overflow. Areas farther from the river (1001 m – 4540 m) are potentially at lower risk of flooding than the closer ones. Land cover was among the top variables with seven types identified: tree cover, shrubland, grassland, cropland, built-up areas, sparse vegetation and water bodies, including herbaceous wetlands, as shown in Figure 2c. A larger percentage of the catchment is covered in shrubland (67.08%), while water bodies and built-up areas, as significant contributors to flood susceptibility, occupy 0.28% and 3.21%, respectively. The natural drainage system was derived from the Strahler stream order (Strahler 1957) in ArcMap 10.8, where five categories were established (Figure 2d). Orders 5 and 4 classify the main river channels, which include the Notwane, Segoditshane and Metsimotlhabe rivers (depicted in Figure 1) as part of the primary rivers, while order 1 identifies small valleys and tributaries. The rivers disperse across the catchment, converging around the Kgatleng region, where Mochudi is located. Figure 2e further illustrates that rainfall concentrations are mainly found in the southern and northeastern parts of the catchment, indicated by high precipitation values (above 484 mm per annum). Moderate to low rainfall is represented by lower values in the central and northern parts of the catchment. However, this does not imply that these areas cannot experience flooding if rain occurs over extended periods or at high intensities. Long-duration rainfall saturates the soil, reducing its ability to absorb additional water, eventually causing surface run-off and flooding, while high-intensity rainfall compacts soil pores and leads to swelling, resulting in more run-off. As a contributing factor, slope also shows the highest recorded values between 28.51 and 55 degrees in the southern and southeastern side of the catchment. These are very steep slopes in hilly upland areas. The lowest slope is primarily found in the central and northern parts of the catchment (Figure 2f) between 0 and 5 degrees, featuring flat and gentle slopes contributing about 7286 km^2^ of the study area.

Soils in Figure 2g present moderate to excessive permeability, such as cambisols, luvisols and arenosols, which are widespread in the central and western parts of the catchment. These soils cover the majority of the area, while clay-rich soils with low water permeability are found in the southern part, covering only 3.8 km^2^. Finally, high and low intensities of water accumulation have been portrayed in Figure 2h. Low to no accumulation of water (indicated by light tones) occupies almost the entire catchment. Moderate to high accumulations (indicated by dark tones) occur mainly at river tributaries within the catchment. Similar to the distance to the river, there is an inverse relationship between the intensity of water accumulation and stream length. The highest accumulations occur downstream around the Mochudi area (7.8 cm), while the lowest accumulations are recorded upstream (0 cm) around Kanye and Lobatse.

### Contributions of flood conditioning factors to flooding over the Notwane Catchment

**FIGURE 3:**
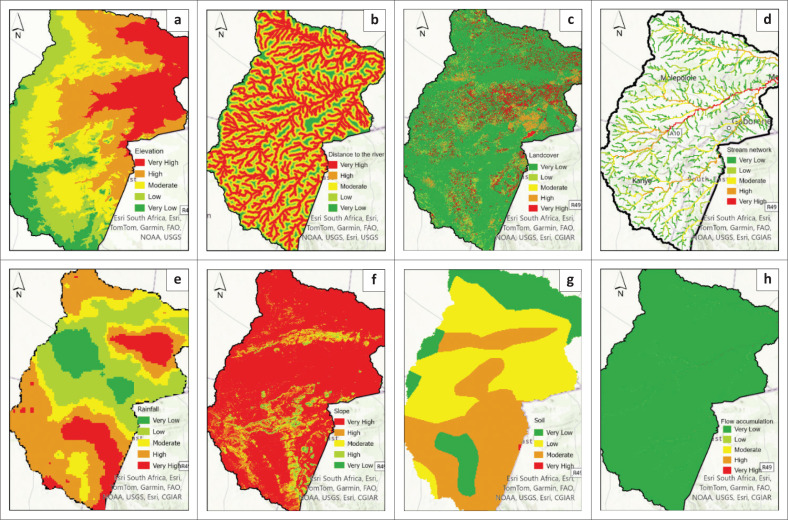
Reclassified susceptibility maps of the flood conditioning factors (FCFs): (a) elevation, (b) distance from the river, (c) landcover, (d) stream networks, (e) rainfall, (f) slope, (g) soil and (h) flow accumulation.

Figure 3a shows elevation susceptibility levels indicating a large domination of the moderate level of flood susceptibility as they cover the largest area in the lower elevations (25.08%, i.e. 2320.63 km^2^), of the catchment, followed by high susceptibility occupying at least 24.13% (2233.19 km^2^). The least susceptible areas are shown by low (19.68%) and very low (12.02%) levels, corresponding to higher elevation areas, hence minimal flood risk. The addition of the most critical susceptibility levels, that is, very high and high, is equivalent to 43.22% of the catchment area being at risk of flooding. Figure 3b indicates an inverse relationship between distance to the river channels and susceptibility. For instance, areas closest to the river (within the 0 m – 500 m distance) cover 34.18% of the total area, the largest proportion, which means 3163.39 km^2^ of the catchment is at the highest risk of flooding because of their proximity to the river. Notably, as the distance from the river increases, the area decreases; for instance, the area between 3001 m and 4025 m (farthest from the river) comprises only 7.54% of the total area. Figure 3c shows the susceptibility levels of land cover, with the total area of the lowest level (very low) covering 7125.015 km^2^, which is the most extensive coverage. Very low and low susceptibility levels of land cover have the lowest average Getis-Ord Gi z-scores of –1.467 and –0.506, respectively. This indicates that there is clustering of areas with low susceptibility, while the highest average GiP-value is observed mostly in the low and moderate susceptibility levels (covering 21.1 km^2^), demonstrating consistent patterns of flood-resilient land covers such as tree cover, shrubland and grassland. On the other hand, high susceptibility (269.99 km^2^) and very high susceptibility cover the second-largest area (1036.96 km^2^) and have the highest average Gi z-scores at 1.17 and 1.90, respectively. Contrarily, the very high susceptibility level results show the lowest average Gi *p*-values of 0.062, indicating that clustering of highly susceptible areas is statistically significant and revealing clear hotspots for flood risk with land covers such as water bodies and built-up areas. Susceptibility levels are further portrayed in the stream networks in Figure 3d. The highest sum of stream length across all susceptibility categories is the very low (2137.6 km) and low (1083 km) categories, comprising stream orders 1 and 2, which explains a dense network, potentially leading to localised flooding during high-intensity rainfall. On the other hand, the 4th and 5th orders (i.e. high and very high susceptibility), at 386.28 km and 158.74 km, respectively, have short total lengths as a result of greater discharge as they collect flood water from several tributaries, leading to heavy river discharge. Rainfall susceptibility levels are also depicted in Figure 3e, with very low susceptibility occupying 8.92% of the total area, while low and moderate levels cover 27.48% and 15.80%, respectively. The largest proportion of the study area falls within high susceptibility, which covers 34.73% of the total area, amounting to about 3214.83 km^2^. This area receives an average total rainfall of above 449.4 mm per annum – 483 mm per annum. The very high (above 484 mm per annum) level of susceptibility occupies 13.07% of the total area, indicating that this area receives high rainfall intensity and therefore has the potential to cause severe flood risk. Very high and high susceptibility of slopes occupy a larger total area of 7286.04 km^2^ and 1317.96 km^2^, respectively (Figure 3f). These areas are primarily low-lying flat and depression areas with a 0–5 degrees slope. The least dominant susceptibility levels are low (167.59 km^2^) and very low (81.74 km^2^), where elevated areas prevail, leading to rapid run-off and very low infiltration capacity. This situation is likely to contribute to flash flooding in nearby valleys and streams, but flooding in these elevated areas is unlikely. Soil susceptibility was classified into four categories, with low to moderate susceptibility contributing to a higher percentage of the catchment area, as shown in Figure 3g. The highest category constitutes 40.9% (low) of the catchment area, comprising luvisols soils; moderate at 36.7% of the total area (cambisols soils) and very low susceptibility, consisting of arenosols soils at 22.35% of the area. These three types of soils have moderate to excessive porosity, characterised by sandy clay loam and loamy sands that slightly retain water, suggesting that these areas are less prone to flooding. A very small part of the catchment has high susceptibility, occupying about 0.04% of the total area, corresponding to vertisols and fluvisols soils. Figure 3h depicts categories of flow accumulation related to flood susceptibility. The Gi* *z*-score and corresponding *p*-values indicate that very high and high flow accumulation areas exhibit strong hotspots and high statistical significance. This is demonstrated by clustering with average Gi *z*-scores of 43.34 and 10.19, and average *p*-values of 0.071 and 0.00, encompassing very small total areas of 2.12 km^2^ and 2.83 km^2^, respectively. Likewise, moderate flow accumulation shows statistically significant clustering (Gi *z*-score of 2.31; average *p*-value of 0.020), covering an area of 3.19 km^2^. Conversely, areas totalling 9237.88 km^2^ and 8.83 km^2^ are categorised as very low to low zones, exhibiting strong cold spots with Gi *z*-scores of –2.32 and –13.59 and average *p*-values of 0.086 and 0.022, respectively, indicating a lower likelihood of experiencing significant flooding.

### Weighting and flood susceptibility mapping over the catchment

#### Pairwise comparison matrix for analytical hierarchy process weighting

Table 2 is a pairwise comparison matrix showing that rainfall is regarded as the most critical factor for flood analysis in the Notwane Catchment, with the highest priority at 0.387 (38.79%) represented as the normalised principal Eigen vector. It is ranked higher than all other factors with values of 5, 2, 5, 5, 6, 1, 5, and 8 when compared to land-use, elevation, flow accumulation, slope, soil type, distance from the river and stream channels, respectively. Land-use is the second most important factor after rainfall, with values of 2, 5, 3, 7, 1/5, 5 and 3, respectively, constituting a weight of 0.213 (21.35%). Furthermore, elevation (0.09), slope (0.062%), distance from the river (0.089) and stream order (0.057) are portrayed as moderate to least important, respectively. Soil type and flow accumulation have smaller priority weights of 0.049 (4.95%) and (4.91%), respectively, indicating that they are considered less critical FCFs than the other factors.

**TABLE 2:** Pairwise comparison matrix of selected flood conditioning factors using Saaty’s scale.

Matrix	Land use/cover	Elevation	Flow accumulation	Slope	Soil type	Rainfall	Distance from the river	Stream channels	Normalized principal eigen vector
Land use/cover	1	2	5	3	7	1/5	5	3	0.213
Elevation	1/2	1	1	1	1	1/2	2	1	0.090
Flow accumulation	1/5	1	1	1	1	1/5	1/3	1/2	0.049
Slope	1/3	1	1	1	1	1/5	1	1	0.062
Soil type	1/7	1	1	1	1	1/6	1/3	1	0.049
Rainfall	5	2	5	5	6	1	5	8	0.387
Distance from the river	1/5	1/2	3	1	3	1/5	1	3	0.089
Stream channels	1/3	1	2	1	1	1/8	1/3	1	0.062

#### Consistency analysis and error calculation

Table 3 shows that pairwise comparisons are consistent and trustworthy with the expected priority weights of the FCFs, with a low ordinal inconsistency Psi of 6% (0.06), CR of 7.8% (0.078) and mean relative error (MRE) of 46.8% (0.46) as recorded in the test.

**TABLE 3:** Consistency analysis and error calculation results from analytical hierarchy process weighting.

Consistency ratio	Values (%)
Psi	6.0
CR	7.8
MRE	46.8

MRE, mean relative error; CR, consistency ratio.

#### Weighted overlay analysis

**FIGURE 4:**
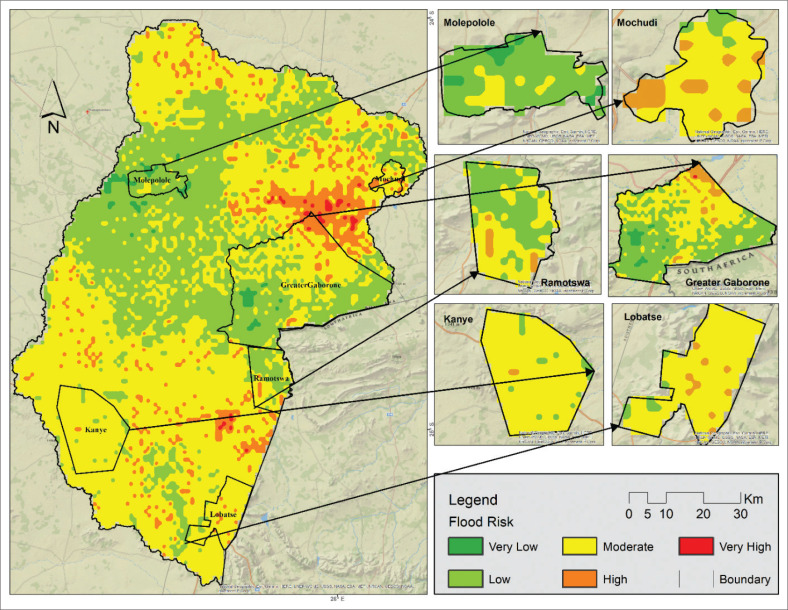
Flood susceptibility map of the Notwane Catchment and its localities.

Figure 4 shows flood susceptibility in the Notwane Catchment area and respective settlements. The map shows that Greater Gaborone exhibits concentrated regions of high to very high flood risk, while nearby locations such as Molepolole, Mochudi, Ramotswa, Kanye and Lobatse generally display low to moderate flood susceptibility with smaller and localised patches of high-risk areas. As shown in Figure 4 and Table 4, Greater Gaborone shows patches of high (5.6%) and very high susceptibility (0.2%) in the central areas. In contrast, most of the area is classified as moderate, as it occupies low to moderate susceptibility levels at 49% and 42.1%, respectively. A geographical influence in the catchment is also recognised in the central and southern parts of the map, showing prominent flood-prone areas (moderate to high susceptibility). At the same time, the northern regions are dominated by very low susceptibility. Molepolole, for instance, depicts low (79.4%) flood susceptibility, indicating low susceptibility across the area covering about 74.1 km^2^. The largest proportion of the area in Mochudi, on the other hand, is characterised by moderate susceptibility, occupying 70.9% of the area, reflecting moderate flood threats, whereas small areas of high susceptibility (10.8 km^2^) are also reflected.

**TABLE 4:** Distribution of flood susceptibility levels across the catchment.

Site	Risk level – area (km^2^)				
	Very low	Low	Moderate	High	Very high
Greater Gaborone	24.6	459.5	391.5	52.0	1.7
Ramotswa	0.0	76.1	73.1	7.0	0.0
Lobatse	0.0	6.8	143.8	6.5	0.0
Kanye	0.0	12.8	306.8	1.4	0.0
Mochudi	0.0	4.5	37.4	10.8	0.0
Molepolole	7.6	74.1	11.7	0.0	0.0
Notwane Catchment	**67.1**	**3077.0**	**5201.2**	**651.5**	**29.7**

Note: Bold values signify the total areas that each susceptibility zone occupy in the catchment.

A substantial part of Ramotswa lies in very low to moderate susceptibility zones, spreading over 48.7% and 46.8%, respectively, shown mainly on the periphery of the settlement. Kanye is primarily covered by moderate susceptibility, which is about 95.6%, or 306.8 km^2^, while low susceptibility areas are scattered across the central parts of the region. The reason for the low susceptibility of these two villages is that they both lie in a generally elevated location. Moderate flood susceptibility levels also accumulate a significant portion (91.5%, 143.8 km^2^) of Lobatse, which has scattered low and high susceptibility areas. Generally, the catchment is at moderate susceptibility of flooding as it is covered by 57.6% of moderate susceptibility, 5201.2 km^2^ of the catchment, followed by low susceptibility covering 3077 km^2^ of the catchment. High (7.2%) and very high (0.3%) susceptibility cover smaller areas, 651.5 km^2^ and 29.7 km^2^, respectively, and are concentrated between the north of Greater Gaborone and the south of Mochudi. Patches of high and very high susceptibility areas are also depicted in the southern parts of the catchment, around the outskirts of Ramotswa, Kanye and Lobatse areas, as demonstrated in Figure 4.

#### Sensitivity analysis of flood conditioning factors

**FIGURE 5:**
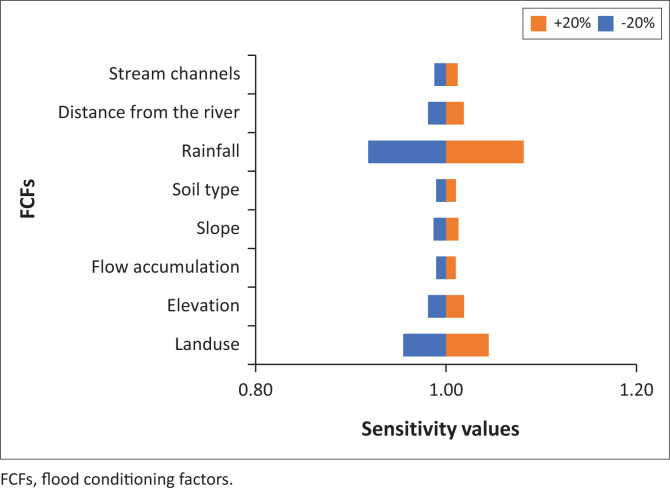
Sensitivity analysis of flood conditioning factors (FCFs) based on (±) 20% variations at 5% intervals.

The tornado plot shown in Figure 5 demonstrates the indices across all factors ranging between 0.92 and 1.08. This means that the model is relatively stable. Rainfall is depicted as the highest variation (SI = 0.92 – 1.08), indicating its most influential nature on the model. Land-use sensitivity variation ranges from 0.96 to 1.04, identified as the second-highest variation. The rest of the other factors, i.e. elevation, soil type, slope, flow accumulation and stream flow, has low variations (ranging from 0.98 to 1.02). This means that these factors have a stable influence, confirming less dependence of the model on them.

## Discussion

This chapter discusses the results derived from the analysis. The study has developed the first comprehensive susceptibility assessment for the urbanising Notwane Catchment using a blended AHP-GIS framework supported by spatial statistics and PCA. This study contributes to existing flood research in Botswana, which has mainly focused on flood variability, impacts, vulnerabilities and adaptive capacity, mostly in Botswana’s wetlands and protected areas (Motsholapheko, Kgathi & Vanderpost 2015; Mpalo, Basupi & Tsidu 2025; Sejie, Mosarwane & Gaogane 2025; Wolski & Murray-Hudson 2008) rather than on spatial susceptibility mapping in urban catchments.

### Spatial patterns of flood susceptibility and implications for flood risk management

Rainfall (38.7%) has been identified as the most influential factor to floods, followed by land-use (21.3%), while selected topographic and hydrological factors, such as elevation, slope, distance to the river and stream channels, have moderate to low contribution. This shows that the hydrological and climatic characteristics of semi-arid catchments are dominated by high rainfall, temporary drainage systems and rapid land-use change (Lindle et al. 2023). Likewise, Aloui et al. (2024), Nigatu et al. (2023) and Ogundolie, Olabiyisi and Ganiyu (2024) added that rainfall has a strong relationship with flood hazard, influencing groundwater recharge and surface run-off, where even moderate amounts can lead to flooding. However, rainfall patterns differ as established by studies in humid and temperate regions, where flow accumulation and drainage density are deemed as more influential than other factors (Nsangou et al. 2022). This affirms that different climate characteristics also contribute towards shaping the prioritisation of FCFs. Short intense rainfalls are often realised in semi-arid environments (Chowdhury 2024; Ibrahim et al. 2023). This study shows that not only short intense rainfall but also prolonged rainfall plays a role in reducing infiltration and increasing soil saturation while continuing surface run-off over extended periods. This is supported by previous studies in Botswana, which identified rainfall with interannual and intra-seasonal fluctuations as the primary driver of flooding (Tsheko 2004; Wolski & Murray-Hudson 2006), corroborating the assignment of high weight to rainfall in the present study for the Notwane Catchment. The spatial distribution of flood susceptibility in the catchment depicts that a concentration of high and very-high susceptibility zones is found in the low-lying areas, particularly downstream of Greater Gaborone, while other settlements such as Molepolole, Mochudi, Ramotswa, Kanye and Lobatse are mostly characterised by low to moderate susceptibility. These zones are observed in areas of high flow accumulation, fitting with the main river channel (Segoditshane, Metsimolhabe and Notwane), hence confirming the relevance of topographical structures on flood production or river overflow. The patterns further reflect changes in land-use intensity, driven by differences in population densities and impervious surface area capacity across the study sites. These effects are increased by changes in land cover in the catchment, as built-up areas and impervious surfaces lead to rapid run-off instead of rainwater being absorbed naturally into the soil. Similar spatial clustering has been observed in other urban catchments, where rapid land-use change increased flooding even under moderate rainfall conditions (Baig, Atif & Tahir 2024; Bibi, Reddythta & Kebebew 2023). These observed patterns highlight that flood susceptibility in the Notwane Catchment is not uniformly distributed but is strongly influenced by the interaction of other factors such as topography, drainage structure and urban expansion. The Notwane Catchment is largely at low to moderate risk of flooding, covering over half of the area (57.6%), which means frequent, but manageable flooding events can still disrupt the daily operations of the communities, if unprepared. This is supported by Paulik et al. (2021), indicating that areas with moderate susceptibility often require significant attention in flood risk management planning and preparedness because, with increased urbanisation and climate change, the risk could be exacerbated. High (7%) and very high-risk (0.3%) zones occupy relatively smaller areas, but it is critical to note that they are concentrated near Greater Gaborone (where the city is located), as well as Mochudi, and southern settlements (Ramotswa, Kanye and Lobatse). These areas are characterised by increased population density and critical infrastructure, making them vulnerable to damaging flood impacts and affecting a larger population, damaging roads, homes and public services (Nabinejad & Schüttrumpf 2023). This, therefore, calls for intentional, site-specific planning while also ensuring prioritisation of high-risk zones and accommodating moderate risk areas into the wider disaster risk reduction framework for informed advisory and decision-making.

The notable flood patterns, coupled with limited historical flood data in the catchment, steered the study towards an AHP-GIS approach that applied community and expert knowledge to identify and rank FCFs in the catchment. This approach is advocated by recent studies within the greater Gaborone area, in the peri-urban Tlokweng village, which underlined that a community-led approach to risk assessments is fundamental (Sejie et al. 2025). This study’s systematic incorporation of community and expert knowledge in identifying and weighing FCFs institutes more practical, inclusive, context specific and effective flood risk management, which can be used as a decision-support tool for disaster management authorities while enabling risk-informed development within data-scarce, semi-arid catchments. Nonetheless, the advances in Artificial Intelligence (AI) and other computational technologies in flood risk mapping, such as machine learning and neural-fuzzy ensemble models are increasingly becoming dominant in data-rich regions (for example, Costache et al. 2020a). These methodologies rely heavily on large datasets and offer limited interpretability (Costache et al. 2020b), which can restrict their practical application in semi-arid regions like Botswana’s Notwane Catchment with insufficient flood data. However, the AHP-GIS method used in this study keeps transparency and straight relevance to local disaster management stakeholders (Caporale & Rinaldi 2025), as it allows for an active participatory weighting of the participants’ self-selected FCFs. Through the utilisation of PCA, Global Getis-Ord Gi* statistics and sensitivity analysis, the study has enhanced the robustness of the method while also warranting that flood susceptibility maps are efficient for disaster risk reduction. This robustness is demonstrated by sensitivity analysis, which shows that the model is moderately stable to changes in FCF weights, with rainfall and land-use constantly having a dominant influence across all scenarios. This is further supported by the consistency analysis of 7.8%, which verifies the credibility of expert judgements. This study, therefore, developed the first flood susceptibility map for the Notwane Catchment, providing baseline information for local disaster management authorities. The study further established the feasibility of a blended AHP-GIS framework in large-scale, limited data and semi-arid catchments, combining expert knowledge, community input and spatial analyses. Finally, the findings convey methodological insights and real-world practical guidance while offering a decision-making support framework that can advise on urban development, disaster preparedness and policy development in semi-arid environments.

## Conclusion

The study concludes that detailed and meaningful flood risk assessments can be produced in data-scarce regions characterised by sparse hydrological monitoring networks, increased flood impacts as a result of climate change and rapid urbanisation made possible by integrating local knowledge with geospatial analysis. This study challenges decision-makers to start institutionalising nature-based solutions, such as preserving and restoring shrubland and tree cover. The study further reveals that the Notwane Catchment has spatial variability of flood susceptibility as a semi-arid region, constituting both urban and peri-urban areas with dominance of moderate susceptibility zones across the catchment. For example, the study shows Greater Gaborone as the most flood-prone area in the catchment while surrounding settlements such as Molepolole and Lobatse experience lower flood susceptibility levels. This study, therefore, recommends the development of targeted flood management strategies that are site specific to address the localised nature of flood susceptibility. This can include rechanneling floodwater through maintained drainage systems, especially in low-lying areas. The study further recommends strengthening the use of geospatial methodologies and machine learning in flood susceptibility assessments to refine predictions and promote susceptibility-informed urban development in the country, prioritising flood mitigation strategies in urban catchments. It also suggests improving statistical clustering techniques to better capture the complex relationship between flood susceptibility levels and enhance prediction in urban catchments. By integrating participatory data, geospatial analysis and spatial statistics, this study fills a critical methodological gap and provides a transferable framework for flood susceptibility assessment in semi-arid, data-limited regions.
